# Increased Aluminum Content in Certain Brain Structures is Correlated with Higher Silicon Concentration in Alcoholic Use Disorder

**DOI:** 10.3390/molecules24091721

**Published:** 2019-05-03

**Authors:** Cezary Grochowski, Eliza Blicharska, Jacek Bogucki, Jędrzej Proch, Aleksandra Mierzwińska, Jacek Baj, Jakub Litak, Arkadiusz Podkowiński, Jolanta Flieger, Grzegorz Teresiński, Ryszard Maciejewski, Przemysław Niedzielski, Piotr Rzymski

**Affiliations:** 1 Department of Anatomy, Medical University of Lublin, Jaczewskiego 4, 20-090 Lublin, Poland; jacek.baj@me.com (J.B.); maciejewski.r@gmail.com (R.M.); 2 Department of Neurosurgery and Pediatric Neurosurgery, Medical University of Lublin, Jaczewskiego 8, 20-954 Lublin, Poland; jakub.litak@gmail.com (J.L.); apodkowinski@wp.pl (A.P.); 3 Department of Analytical Chemistry, Medical University of Lublin, Chodźki 4a, 20-093 Lublin, Poland; bayrena@o2.pl (E.B.); jolanta.flieger@umlub.pl (J.F.); 4 Department of Clinical Genetics, Medical University of Lublin, Radziwiłłowska 11, 20-080 Lublin, Poland; jacekbogucki@wp.pl; 5 Faculty of Chemistry, Department of Analytical Chemistry, Adam Mickiewicz University in Poznań, 89B Umultowska Street, 61-614 Poznan, Poland; jed.proch@gmail.com (J.P.); pnied@amu.edu.pl (P.N.); 6 Department of Forensic Medicine, Medical University of Lublin, 8b Jaczewskiego St, 20-090 Lublin, Poland; mierzwinska.aa@gmail.com (A.M.); grzegorz.teresinski@umlub.pl (G.T.); 7 Department of Environmental Medicine, Poznan University of Medical Sciences, 61-701 Poznan, Poland; rzymskipiotr@ump.edu.pl

**Keywords:** aluminum, silicon, ICP-OES, trace elements, brain trace element concentration, brain toxicity

## Abstract

Introduction: Alcohol overuse may be related to increased aluminum (Al) exposure, the brain accumulation of which contributes to dementia. However, some reports indicate that silicon (Si) may have a protective role over Al-induced toxicity. Still, no study has ever explored the brain content of Al and Si in alcoholic use disorder (AUD). Materials and methods: To fill this gap, the present study employed inductively coupled plasma optical emission spectrometry to investigate levels of Al and Si in 10 brain regions and in the liver of AUD patients (*n* = 31) and control (*n* = 32) post-mortem. Results: Al content was detected only in AUD patients at mean ± SD total brain content of 1.59 ± 1.19 mg/kg, with the highest levels in the thalamus (4.05 ± 12.7 mg/kg, FTH), inferior longitudinal fasciculus (3.48 ± 9.67 mg/kg, ILF), insula (2.41 ± 4.10 mg/kg) and superior longitudinal fasciculus (1.08 ± 2.30 mg/kg). Si content displayed no difference between AUD and control, except for FTH. Positive inter-region correlations between the content of both elements were identified in the cingulate cortex, hippocampus, and ILF. Conclusions: The findings of this study suggest that AUD patients may potentially be prone to Al-induced neurodegeneration in their brain—although this hypothesis requires further exploration.

## 1. Introduction

Aluminum (Al), the most abundant metal and third most common element in the Earth’s crust, is increasingly used for various purposes in a number of sectors within the pharmaceutical (e.g., as antacids, phosphate binders, buffered aspirins, adjuvant) cosmetics (e.g., in antiperspirants) and food (e.g., as a packing material, food additive) industries. The majority of Al is continuously extracted from existing ores, with recycling processes accounting only to 40% of its overall supply [1]. This results in a rise in environmental and circulating levels of Al, and as estimated, its exposures have increased at least 30-fold over the last 50 years—with a mean of 11 kg of Al being currently refined for every human, annually [2].

There is no specific biological role identified for Al, and its ion (Al^3+^) is known to reveal toxic action. Accumulating evidence from in vitro and in vivo experimental studies, as well as epidemiological observations, demonstrate that increased exposures to Al can lead to a number of adverse health effects [3]. Al has been postulated to induce oxidative stress in various cell types [4,5], interfere with estrogen receptors [6], support osteomalacia via phosphate deficiency, impair calcium uptake and engender dysfunctional osteoblast proliferation [7], as well as to alter iron homeostasis by disrupting intestinal Fe absorption and normal tissue ferritin levels [8]. Of highest concern is that studies on Al uptake have revealed a neurotoxic action that is potentially implicated in different neurodegenerative disorders, including encephalopathy, Alzheimer’s disease and multiple sclerosis [9,10,11]. As shown in experimental animals, Al can lead to accumulation of Aβ and tau protein, and induction of neuronal apoptosis in the brain [12,13], and impair learning and memory functions [14,15]. Acute exposures to Al in human were associated with cognitive impairment, such as agitation, confusion, or myoclonic jerk [16,17], while occupationally exposed subjects revealed disruption in memory and concentration [18,19]. Thus, research has assessed its content in human bones [20,21], in organs, such as the brain [22] and uterus [23,24], as well as in fluids including urine [25], serum [26], breast milk [27], and semen [28].

A number of activities are known to significantly increase Al exposure. These include specific industrial and agriculture occupations, first-hand and second-hand smoking, and the use of recreational drugs. such as heroin or cocaine. Furthermore, selected food products have been shown to have high Al content, e.g., jellyfish, fried twisted cruller, or microalgal supplements if Al compounds were used to harvest the biomass [29,30,31]. Al is known to be poorly absorbed in the gastrointestinal tract at levels of 0.1–1.0% of the oral dose, and in healthy subjects, most of the absorbed pool is readily excreted from the body in the urine. However, some factors, such as consumption of citric acid in the form of fruit juices, markedly increase Al absorption. Hence, under sustained exposure of the gastrointestinal tract, or/and under certain conditions, particularly renal failure, increased Al accumulation in the body can occur. This effect is particularly noticeable in the central nervous system [32].

Some additional factors, such as excessive consumption of ethanol, have been suggested to increase Al bioavailability due to increased permeability of the intestinal mucosa [33]. However, experimental studies have shown that silicon (Si) may be protective against aluminum accumulation in the brain [34,35]. It has been previously hypothesized that alcoholic amnesia and dementia may also arise from increased Al exposures in individuals through the excessive consumption of ethanol beverages [33] and that Al accumulation may be regulated by Si availability. Thus, the aim of the present study is to compare Al and Si content in different regions of the brain of individuals with alcohol use disorder (AUD) and in control subjects and to assess whether levels of these two elements in the brain and liver reveal any association. To the best of our knowledge, this is the first, albeit preliminary study to demonstrate that AUD patients may face increased Al accumulation in their brains.

## 2. Results

### 2.1. Demographic Characteristic

The studied AUD and control group consisted of 31 and 32 subjects, respectively. Their demographic data are summarized in Table 1.

### 2.2. Al Content

The total (mean ± SD) content of Al in the brain of the AUD subjects was 1.59 ± 1.19 mg/kg. Al was identified in every studied area, albeit at levels decreasing in the following order: FTH > ILF > INS > SLF > ACC > CA > HPC > PCG > NAc > PFC. All control samples displayed Al content below detection limits. The Al levels in the liver displayed no significant difference between AUD and control subjects (Table 2).

A number of significant, mostly positive correlations in Al content in various brain regions of the AUD subjects were identified. INS content was, however, negatively correlated with PFC and ILF. Additionally, the liver levels of Al revealed a positive correlation with that found in PFC, PCG, FTH, SLF, and ILF (Table 3).

### 2.3. Si Content

Si was identified above detection limits at varying frequency in all brain regions of both AUD and control subjects at total mean ± SD content of 32.1 ± 11.3 and 5.6 ± 2.6 mg/kg, respectively (*p* < 0.001, Mann-Whitney U test). In both groups, Si levels displayed high variance of observed levels. In AUD subjects, Si mean content decreased in the following order: FTH > INS > ACC > CA > PCG > SLF > NAc > HPC > ILF > PFC, while in control, a strikingly different order was observed: PFC > SLF > ACC > PCG > HPC > NAc > FTH > INS > CA > ILF. However, the only significant difference in Si content between the AUD and the control group was found for FTH. Liver content of Si did not differ between groups (Table 4).

A number of significant correlations in Si content in various brain areas were identified in the AUD group. Additionally, Si levels in the PFC, PCG, ACC, CA, and FTH were positively correlated with that found in the liver (Table 5).

In the control group, Si content in all brain areas was significantly and positively intercorrelated. A similar observation was made for Al content in the liver, and that found in every studied brain area (Table 3). The conducted analysis revealed the existence of a series of (22) positive, statistically significant r-Spearman correlation coefficients, attesting to the existence of a positive correlation. The highest value of the correlation coefficient was observed in the correlation between the amount of silicon in the Si-PCG and the Si-ACC. In the liver, four statistically significant positive correlation coefficients were noted—with the Si-CA, Si-FTH, Si-SLF, and Si-ILF (Table 6).

There were also two statistically significant, negative correlation coefficients seen, indicating the existence of a negative correlation between the amount of silicon in the Si-PFC and Si-NAc and Si-INS. Likewise, a number of correlations in Si content in various areas were identified in the control group.

### 2.4. Association Between Al and Si Content

Several significant positive correlations between Al and Si content in the different brain regions of AUD subjects were identified (Table 7). Inter-region correlations were found for ACC, HPC, and ILF. The liver content of both elements was also found to be significantly associated.

## 3. Discussion

This is the first study to demonstrate that Al content is significantly increased in the brains of AUD subjects. Considering that Al has been implicated in neurodegenerative processes, this appears to be a clinically relevant observation. As noted previously, chronic Al exposure can cause the accumulation of AβP, impair spatial learning memory and induce conformational changes to proteins related to neurodegeneration. These include tau, PHF-tau, synuclein, amylin, ABri, microglobulin, and APP [36,37,38,39,40,41]. A recent study reported brain Al levels of subjects suffering from Alzheimer’s disease and multiple sclerosis, the content of which is in excess of 10 and 50 mg/kg, respectively [11]. The present study found that compared to control, mean brain content of Al in AUD subjects was by order of magnitude higher and exceeded 7 mg/kg.

Studies on animal models have demonstrated the neurotoxicity of chronic exposure to low doses of aluminum. This causes brain aging acceleration through the induction of inflammatory processes, such as an increase in inflammatory cytokines and amyloid precursor proteins, as well as enhanced glial activation. There are several theories explaining the toxicity of aluminum in relation to the brain tissue. It is suggested that its insoluble complexes stimulate the activation of glial cells and magnify the activity of macrophages. These effects were confirmed by studies in rats in which deposits of the element within the striatum and associated renal gliosis were observed [42]. Within the striatum, excessive proliferation of astrocytes and microglia was found in patients with chronic renal failure who had used aluminum gels [43]. Abou-Donia’s research also suggests the direct toxicity of the element to the brain tissue. Such conclusions were drawn while treating dialysis encephalopathy with deferoxamine [44]. In a study conducted by Petrik et al. on a mouse model where aluminum-containing adjuvants were injected, inflammation and loss of cells within the motor cortex and spinal cord were detected, and memory deficits were described [45]. Symptoms of encephalopathy have also been observed among aluminum industry workers [46]. Moreover, progressive cognitive dysfunction, as well as ataxia, dysarthria, and seizures, have been noted among people taking drugs through the intravenous route caused by the preparation of a methadone solution in an aluminum dish [47].

During the consumption of water containing elevated concentrations of this element, significant deficits in cognitive functions were detected among the population [48]. This was confirmed also in the animal model, where behavioral disorders [49], as well as changes in cognitive and morphological functions in the central nervous system, were demonstrated [50].

Memory impairment due to aluminum poisoning was first described in 1921. This was subsequently confirmed by Rondeau et al., who in a 15-year cohort study, demonstrated that daily intake of high doses of aluminum correlates with an increased risk of cognitive decline and dementia. Herein, Al intake brought about the death of neurons and glial cells, and, consequently, deficits in spatial memory, emotional deficits, and impaired memory and learning processes [51].

There are many papers reporting an increased risk of developing Alzheimer’s disease by drinking water with a high concentration of aluminum. McLachlan et al., in their study, showed that people living in areas where the concentration of this element in water was above 100 μg/l have a higher risk of suffering from Alzheimer’s disease [50], which was also confirmed by a study by Rondeau et al. [51]. A meta-analysis by Flaten [52], as well as a study carried out after 15 years on a large population, confirmed the association of the development of Alzheimer’s disease with high aluminum content in drinking water [51].

Increased Al content in AUD subjects leads to the hypothesis that this metal could plausibly add to neurodegeneration processes involved in alcoholic amnesia and dementia in subjects over-consuming ethanol. This could partially be due to increased absorption of Al in the gastrointestinal tract due to altered permeability of intestinal mucosa [33] and/or its increased retention because of renal failure, both of which have been observed in AUD subjects [53]. One should further note that various alcoholic beverages can contain detectable content of Al. Herein, the highest values are found in red and white wine [54] by way of its binding to tartaric acid and other organic acids that can increase the bioavailability of the element [55]. As suggested by some authors, Al content in wine should not exceed 0.5 mg/L. Increased Al content was also found in beers stored in aluminum cans [56]. It is thus plausible that increased Al accumulation in the brain of AUD subjects results from ethanol-induced binge intake, as well as from general elevated oral exposures to this metal.

In addition, the present study also investigated brain Si content.

Silicic acid has not been found to interfere with organic molecules in biological systems. However, it is able to react with Al_3_^+^ (at pH ≥ 5). As found, there was no statistically significant difference in Si content in brain regions between AUD and control subjects, with the exception of FTH. Previous research has shown that AUD subjects display positive inter-region correlations between Al and Si levels in ILF, HPC, and ACC. It remains unknown whether Si may immobilize Al in these areas or alter its toxicity—these hypotheses require further exploration in experimental models. Silicic acid has not been found to interfere with organic molecules in biological systems. However, it is able to react with Al^3+^ (at pH ≥ 5).

The daily intake of Al is about 20mg, and silicon absorption has been evaluated at 20–50 mg. Water and other drinks can provide 19% of daily intake [36].

Using an Atlantic salmon model, Exley et al. were the first to suggest the protective effect of silicon in Al. As demonstrated, fish exposed to water with high Al concentration and low Si concentration were observed to be intoxicated. Moreover, whole-body Al content was assessed as being higher in those fishes [57]. And some additional studies report that the greater amount of absorbed Al was of a temporary situation, some Al was permanently stored in tissue. This resulted in a delay before excretion [58]. The preliminary work by Belia and Roberts claims that an increased intake of silicic acid can mobilize Al and decrease the tissue storage concentration [59]. Moreover, studies conducted by Desouky et al. in which the accumulation of Al in snail cells was investigated, revealed co-accumulation of Si, even without exogenous Si supplementation [60].

Although silicic acid was noted to reduce Al uptake in the intestine [61], there are several different routes of Al uptake—dermal, olfactory and respiratory. White et al. report that Si-Al interactions occur within the tissues in vivo [62]. They also concluded that, in spite of normalization of Al levels after nine days, there were no behavioral improvements at day 15, which suggest long-term Al effects on nervous tissue. Currently, there are no known mechanisms of Si transportation into the tissue, however, because of the small dimensions of Si(OH)_4_ and its neutral charge it is possible for silicic acid to enter into the cells through passive diffusion. Moreover, chronic alcohol consumption causes changes in cytoskeletal and tight junction assembly, thus the dysfunction of blood-brain barrier and increased porosity.

In our study, increased Si levels accompanied increased Al levels in three out of 10 analyzed brain structures. Herein, the Si concentration levels were several times higher than Al levels. Of note, studies report high Si concentration in beer [63], which may have protective properties in this group.

However, while these findings imply that AUD patients may be more prone to Al-induced toxic effects in the brain, it should be underlined that the Al content was not explored here in the context of neurodegenerative changes in brain regions or cognitive function impairment. This would require experimental in vivo research and/or intravital neuroimaging of Al in the brain, neither of which has been done. Moreover, the nature of Al and Si association in selected brain regions in AUD remains unknown, and whether the latter may affect the activity of the former is purely speculative.

## 4. Material and Methods

### 4.1. Subjects

The research material consisted of brain and liver tissue samples taken from individuals reported to the autopsies in the Department of Forensic Medicine at the Medical University of Lublin in Poland. Samples were collected from 31 subjects (23 male, 8 female) with AUD, with inclusion criteria of chronic alcohol abuse in their medical history, no history of mental disorders and alcohol level > 2‰ in blood confirmed at the time of the section. Samples were also collected from 31 control subjects (21 male and 10 female). The inclusion criteria for this group were the absence of documented alcoholic and neurodegenerative disorder history, macroscopically unaltered brain tissue, and blood alcohol level of <2‰ as confirmed at the time of the section. The competent prosecutor’s office consented to the collection of tissues, and the study was approved by the Local Bioethical Committee of the Medical University of Lublin (approval No. KE-0254/2018). In addition, the work described in this article has been carried out in accordance with The Code of Ethics of the World Medical Association (Declaration of Helsinki) for experiments involving humans; Uniform Requirements for manuscripts submitted to Biomedical journals.

### 4.2. Sample Tissue Collection and Procedure

Samples were collected by qualified pathologists in accordance with the analytical protocol. In order to prevent contamination of the tissue sample, all materials in contact with the samples were previously decontaminated with 5% (*v*/*v*) suprapure nitric acid solution and thoroughly washed with ultrapure water (Milli-Q, Millipore, Raleigh, NC, USA, resistivity 18.2 MΩ·cm).

After removing the brain from the cranium, the excess of the blood was thoroughly washed with ultrapure water. Meninges were removed with plastic tweezers, and the brain tissue was washed again with ultrapure water to minimize samples contamination with blood or cerebrospinal fluid.

For analysis, 0.5 g of tissue samples were harvested in ten anatomical locations using disinfected plastic knives. Ten areas of the brain were selected: frontal cortex (Broadmann area no. 11, PFC), postcentral gyrus (Broadmann area no. 1, PCG), dorsal anterior cingular cortex (Broadmann area no. 32, ACC), the foot of the hippocampus (HPC), the head of the caudate nucleus (CA), the frontal part of the thalamus (FTH), superior longitudinal fasciculus (SLF), inferior longitudinal fasciculus (ILF) and the nucleus accumbens (NAc), frontal part of the insula (INS).

Additionally, liver samples were collected by dissecting 0.5 g from the 6th segment. All tissue samples were thoroughly rinsed with deionized water and drained on sterile blotting paper. All of the collected samples were weighed. The tissue sample was then put into sterile polypropylene containers (Bionovo), and, afterwards, the initial decay of the organic matrix through the use of 2 mL of 65% suprapure HNO_3_ was performed. The mass loss of the samples was limited, and samples were digested directly after sampling without preliminary drying.

In the last stage of the experiment, each sample was quantitatively transferred to close Teflon containers and digested at 180 °C utilizing the microwave digestion system Mars 6 (CEM, Matthews, NC, USA). After digestion, samples were diluted with water to meet a total volume of 10.0 mL using scaled test-tubes.

### 4.3. Analytical Procedures

The inductively coupled plasma optical emission spectrometer Agilent 5110 ICP-OES (Agilent, Santa Clara, CA, USA) was employed for Al and Si determination. The synchronous vertical dual view (SVDV) of the plasma was accomplished by using dichroic spectral combiner (DSC) technology. This allows axial and radial view analysis simultaneously. In doing so, radio frequency (RF) power was 1.2 kW, nebulizer gas flow—0.7 L min−1, auxiliary gas flow—1.0 L min−1, plasma gas flow—12.0 L min−1, charge coupled device (CCD) temperature was −40 °C, viewing height for radial plasma observation was 8 mm, while accusation time was 5 s. The analysis was repeated three times. The following wavelengths were applied: Al—396.152 nm, Si—288.158 nm. ICP commercial analytical standards (Romil, Cambridge, UK) were used for calibration. Detection limits were determined through 3-sigma criteria and were on the level of 0.01 (mg/kg) wet weight (*w/w*) for all elements determined. The uncertainty for the complete analytical process (including sample preparation) was at the level of 20%. Traceability was assessed by comparison with reference materials. A recovery of 80–120% was considered acceptable for all the elements determined.

### 4.4. Statistical Analysis

All statistical analyses were performed with the use of Statistica v.13.3 (StatSoft Polska Sp. zo.o., Kraków, Poland). The assumption of Gaussian distribution was not met (*p* < 0.05, Shapiro-Wilk test), thus non-parametric methods were then applied. Herein, differences between two and three independent groups were assessed via the Mann-Whitney U test, Pearson’s chi^2^ and Kruskal-Wallis ANOVA, respectively. The correlation between two variables was evaluated with Spearman Rs coefficient. A *p*-value of *s* < 0.05 was considered to be statistically significant.

## 5. Conclusions

The present study demonstrated for the first time that AUD patients are characterized by increased Al content in various brain regions, with the highest content identified in FTH, ILF, and INS. Considering that Al has neurodegenerative potential, and cognitive impairments in alcoholics are linked to ethanol-induced neurodegeneration and alcohol dependence, these findings appear to be clinically relevant. Furthermore, we saw that AUD patients did not display significantly increased levels of brain Si, an element previously postulated to have a protective role over toxicity expressed by Al. Thus, an association between both elements in ACC, HPC, and ILF was observed—the nature of these correlations remains, however, yet to be explored. Our findings are in favor of a hypothesis that has been previously put forward that alcohol overuse may contribute to increased Al exposure and that this element may potentially add to the neurodegenerative outcomes observed in AUD patients, such as dementia. Further research is required to investigate the mechanisms of neurotoxicity of Al under ethanol exposure and to explore whether Al accumulation can be decreased in AUD subjects.

## Figures and Tables

**Table 1:** Demographic characteristic of studied subjects enrolled in this study.

Parameter	Control (*n* = 32)	AUD (*n* = 31)	*p*-Value
Age [years] (mean ± SD)	49.4 ± 19.5	47.9 ± 13.1	0.74
Sex [n/%]			
Female	12 (37.5%)	8 (25.8%)	0.32
Male	20 (62.5%)	23 (74. 2)	
Weight [kg] (mean ± SD)	79.4 ± 21.3	77.9 ± 15.7	0.82
BMI [kg/m^2^] (mean ± SD)	26.7 ± 5.6	26.8 ± 6.2	0.65

**Table 2:** Content of Al in alcohol use disorder (AUD) (*n* = 31) and control (*n* = 32) subjects in different brain structures and in the liver (mg/kg).

		% < LOD	N > LOD	Mean	± SD	Median	Min	Max	CV	*p*-Value
**PFC**	AUD	54.8	14	0.49	0.44	0.27	0.01	1.4	0.20	-
	Control	96.9	1	-	-	-	-	-	-	
**PCG**	AUD	45.1	17	0.95	1.5	0.46	0.04	6.6	2.4	-
	Control	96.9	1	-	-	-	-	-	-	
**ACC**	AUD	41.9	18	1.0	1.3	0.54	0.03	5.0.	1.8	-
	Control	100	0	-	-	-	-	-	-	
**HPC**	AUD	67.7	10	0.98	0.59	0.98	0.08	1.9	0.34	-
	Control	96.9	1	-	-	-	-	-	-	
**CA**	AUD	54.8	14	0.99	1.5	0.39	0.04	5.7	2.2	-
	Control	96.9	1	-	-	-	-	-	-	
**FTH**	AUD	51.6	15	4.0	12.7	0.31	0.04	49.7	161.	-
	Control	96.9	1	-	-	-	-	-	-	
**SLF**	AUD	58.1	13	1.1	2.3	0.21	0.01	8.3	5.3	-
	Control	96.9	1	-	-	-	-	-	-	
**ILF**	AUD	51.6	15	3.5	9.7	0.41	0.02	37.4	93.5	-
	Control	93.7	2	-	-	-	-	-	-	
**NAc**	AUD	71.0	9	0.65	0.79	0.30	0.05	2.4	0.63	-
	Control	90.6	3	-	-	-	-	-	-	
**INS**	AUD	74.	8	2.4	4.1	0.40	0.05	12.1	16.8	-
	Control	100	0	-	-	-	-	-	-	
**Liver**	AUD	51.6	15	1.5	3.0	0.42	0.02	12.0	9.0	0.173
	Control	81.2	16	0.31	0.22	0.30	0.06	0.92	0.09	

**Table 3:** Spearman correlation coefficient for Al content in various brain structures and the liver of AUD subjects. Asterisks indicate *p* < 0.05 analysis of Al concentration. Correlation coefficients marked with * are significant at *p* < 0.05.

	Al-PFC	Al-PCG	Al-ACC	Al-HPC	Al-CA	Al-FTH	Al-SLF	Al-ILF	Al-liver	Al-NAc	Al-INS
Al-PCG	0.43 *										
Al-ACC	0.283	0.574 *									
Al-HPC	−0.049	0.269	0.071								
Al-CA	0.627 *	0.291	0.340	0.021							
Al-FTH	0.393 *	0.339	0.167	0.084	0.561 *						
Al-SLF	0.265	0.585 *	0.178	0.233	0.068	0.505 *					
Al-ILF	0.529 *	0.419 *	0.402 *	−0.323	0.526 *	0.238	0.073				
Al-liver	0.406 *	0.632 *	0.277	0.239	0.349	0.421 *	0.556 *	0.511 *			
Al-NAc	−0.194	0.185	−0.106	0.377 *	0.087	−0.013	0.257	−0.146	0.083		
Al-INS	−0.414 *	0.155	−0.032	0.403 *	−0.158	−0.118	0.124	−0.417*	−0.024	0.655 *	

**Table 4:** Content of Si in AUD (*n* = 31) and control (*n* = 32) subjects in different brain structures and in the liver (mg/kg).

		% < LOD	N > *LOD*	Mean	± SD	Median	Min	Max	CV	*p*-Value
**PFC**	AUD	35.5	20	13.5	37.9	2.5	0.02	171.	1439.	0.474
	Control	43.7	18	11.6	1.5	30.2	0.03	125.	911.	
**PCG**	AUD	12.9	27	31.1	73.1	4.4	0.02	367.	5351.	0.164
	Control	53.1	15	5.9	3.1	9.9	0.11	39.8	97.5	
**ACC**	AUD	9.7	28	40.8	129.	3.9	0.12	683.	16738.	0.326
	Control	65.6	11	6.5	1.8	8.1	0.12	20.2	65.1	
**HPC**	AUD	41.9	18	25.9	47.2	3.8	0.37	183.	2231.	0.076
	Control	50.0	16	5.2	1.8	7.3	0.13	26.8	54.1	
**CA**	AUD	25.8	23	34.9	94.1	2.4	0.12	436.	8856.	0.094
	Control	56.2	14	3.2	1.5	6.2	0.11	24.2	38.7	
**FTH**	AUD	25.8	23	51.1	151.	3.4	0.03	532.	22698.	**0.020**
	Control	50.0	16	3.7	1.0	6.5	0.02	23.6	42.1	
**SLF**	AUD	29..0	22	27..0	53..7	4..2	0..13	225..	2880..	0..887
	Control	68..7	10	8.0..	2..8	10..5	0..19	32..9	111..	
**ILF**	AUD	29..0	22	22.0	43..9	5..8	0..04	192..	1931..	0..149
	Control	56.250	14	2.5	2.2	2.4	0.06	9.7	5.7	
**NAc**	AUD	41.9	18	26.1	80.8	1.8	0.02	341.	6526.	0.759
	Control	53.1	15	5.0.	2.3	6.7	0.24	19.0	45.1	
**INS**	AUD	41.9	18	50.0	127.	4.5	0.12	506.	16264.	0.149
	Control	40.6	19	3.6	1.62	4.9	0.09	17.1	24.2	
**Liver**	AUD	12.9	27	31.3	115.	2.6	0.14	598.	13322.	0.096
	Control	18.7	26	6.0	1.9	17.3	0.07	89.2	299.9	

**Table 5:** Spearman correlation coefficient for Si content in various brain structures of AUD subjects and in their liver. Asterisks indicate *p* < 0.05.

	Si-PFC	Si-PCG	Si-ACC	Si-HPC	Si-CA	Si-FTH	Si-SLF	Si-ILF	Si-liver	Si-NAc	Si-INS
Si-ACC	0.150										
Si-HPC	0.285	0.788 *									
Si-CA	−0.338	−0.036	−0.043								
Si-FTH	0.316	0.632 *	0.717 *	−0.040							
Si-SLF	0.167	0.429 *	0.457 *	−0.008	0.640 *						
Si-ILF	−0.088	0.464 *	0.315	0.104	0.274	0.506 *					
Si-liver	0.467 *	0.624 *	0.742 *	−0.078	0.694 *	0.422 *	0.289				
Si-NAc	0.208	0.244	0.266	−0.043	0.393 *	0.405 *	0.530 *	0.385 *			
Si-INS	−0.579 *	−0.104	−0.145	0.180	0.022	0.032	0.026	−0.302	−0.066		
Si-ACC	−0.490 *	0.095	0.024	0.288	−0.034	0.222	0.378 *	−0.164	0.184	0.581 *	
Total Si	0.067	0.767 *	0.706 *	0.158	0.691 *	0.615 *	0.656 *	0.525 *	0.507 *	0.038	0.333

**Table 6:** Spearman correlation coefficient for Al content in various brain structures and the liver of AUD subjects. Asterisks indicate *p* < 0.05.

	Si-PFC	Si-PCG	Si-ACC	Si-HPC	Si-CA	Si-FTH	Si-SLF	Si- ILF	Si-liver	Si-NAc	Si-INS
Si-PCG	0.719 *										
Si-ACC	0.405 *	0.538 *									
Si-HPC	0.632 *	0.647 *	0.510 *								
Si-CA	0.733 *	0.824 *	0.611 *	0.758 *							
Si-FTH	0.553 *	0.510 *	0.350 *	0.421 *	0.556 *						
Si-SLF	0.385 *	0.370 *	0.283	0.261	0.422 *	0.608 *					
Si-ILF	0.470 *	0.623 *	0.431 *	0.521 *	0.705 *	0.447 *	0.584 *				
Si-liver	0.590 *	0.515 *	0.249	0.288	0.457 *	0.364 *	0.452 *	0.494 *			
Si-NAc	0.669 *	0.693 *	0.515 *	0.489 *	0.661 *	0.642 *	0.573 *	0.599 *	0.512 *		
Si-INS	0.502 *	0.673 *	0.542 *	0.569 *	0.648 *	0.426 *	0.332	0.613 *	0.396 *	0.507 *	
Total Si	0.762 *	0.781 *	0.488 *	0.681 *	0.767 *	0.763 *	0.618 *	0.678 *	0.502 *	0.783 *	0.717 *

**Table 7:** Spearman correlation coefficient calculated for Al and Si content in different brain regions of AUD subjects.

AUD Group											
Si	Al										
	PFC	PCG	ACC	HPC	CA	FTH	SLF	ILF	NAc	INS	Liver
**PFC**	0.379	−0.055	0.437	−0.200	−0.013	−0.163	0.033	0.720	0.485	−0.800	0.045
**PCG**	0.608	0.267	0.541	0.672	0.235	−0.207	−0.219	0.349	0.200	−0.214	0.050
**ACC**	0.582	0.094	0.581	0.783	0.379	−0.019	−0.097	0.460	0.238	−0.107	0.274
**HPC**	0.381	−0.063	0.783	0.666	−0.006	0.233	−0.133	−0.187	−0.095	0.285	0.230
**CA**	0.604	0.191	0.438	0.500	0.481	−0.157	−0.090	0.661	0.714	0.250	0.225
**FTH**	0.836	0.142	0.287	0.607	−0.063	0.461	0.200	−0.081	−0.371	0.600	0.482
**SLF**	0.054	0.217	0.100	−0.116	−0.172	0.327	0.296	0.524	−0.500	0.750	0.112
**ILF**	0.223	0.279	0.502	0.321	0.167	−0.159	−0.466	0.560	0.392	−0.085	0.055
**NAc**	0.000	0.154	−0.218	−0.095	−0.023	−0.200	0.533	−0.023	−0.100	0.035	0.761
**INS**	0.485	0.442	0.600	0.428	0.428	0.333	0.714	0.285	−0.404	0.595	0.216
**Liver**	0.293	0.342	−0.007	0.309	−0.188	0.138	0.216	0.132	−0.190	0.142	0.678
